# Pooling Time Series Based on Slightly Different Questions About the Same Topic Forty Years of Survey Research on Happiness and Life Satisfaction in The Netherlands

**DOI:** 10.1007/s11205-015-0898-5

**Published:** 2015-02-21

**Authors:** Tineke DeJonge, Ruut Veenhoven, Wim Kalmijn, Lidia Arends

**Affiliations:** 1 Erasmus Happiness Economics Research Organization, Erasmus University Rotterdam, Rotterdam, The Netherlands; 2 North-West University, Potchefstroom, South Africa; 3 Institute of Psychology, Erasmus University Rotterdam, Rotterdam, The Netherlands; 4 Erasmus MC, Biostatistics, Rotterdam, The Netherlands

**Keywords:** Rating scales, Comparability, Beta distribution, Reference distribution, Happiness, life satisfaction, Pooling time series

## Abstract

Survey research on subjective wellbeing in The Netherlands started in the early 1970s. The time series happiness and life satisfaction that have emerged since then are unfortunately based on slightly different survey items of which one part uses verbal response scales and another part uses numerical response scales. The diversity of the survey items and a number of other measurement issues, such as the effects of changes in survey mode, hamper comparison over time and make it difficult to establish whether life became any better over the last forty years. These problems can be tackled using the recently developed Reference Distribution Method with which responses to equivalent but not identical survey questions can be pooled to obtain long, consistent time series. We applied the Reference Distribution method to pool time series of happiness and life satisfaction. We conclude that in the past 40 years the Dutch have become slightly happier and satisfied with their lives.

## Introduction

Happiness and life satisfaction have been measured periodically in survey studies since the 1970s in The Netherlands. It was common practice to use questions which had verbal response scales. The present trend however, is to use 10- or 11-point numerical scales with only the anchor points^1^ defined by verbal labels for evaluative measures because they minimise categorisation effects, improve data analysis and produce more reliable data (Scherpenzeel 1999; Voorpostel et al. 2009; OECD 2013; van Beuningen et al. 2014). The variability in response scales used over time impedes the comparability of survey results. An overview of this comparability problem and methods to tackle this is given in DeJonge et al. (2014a). In the current paper^2^ we will elaborate on this work with special attention for the correction of discontinuities in time series.

### The Concepts ‘Happiness’ and ‘Satisfaction with Life’

The terms ‘happiness’ and ‘satisfaction with life’ are no unambiguous concepts. In the social sciences happiness is commonly focused on in the sense of subjective enjoyment of one’s life as a whole (Veenhoven 1984). In this definition ‘happiness’ is synonymous with ‘life satisfaction’. Other researchers though, conclude that these terms do tap different aspects of subjective well-being. Among them are McKennel and Andrews (1980), Saris and Andreenkova (2001), Fischer (2009), and Diener et al. (2010). These authors suggest that happiness is more emotionally driven than life satisfaction and vice versa that life satisfaction is to a larger extent driven by economic conditions than happiness. We however, do not intend to add a contribution to this discussion in this paper but thought it might be helpful to mention some references to papers that do discuss the definition of both concepts. The study presented in this paper was conducted with the purpose to deal with the distortion of trends when making time series of results from different surveys on the same topic comparable and for this purpose we made use of time series of evaluative measures on happiness and life satisfaction. Since the study was of a methodological nature, we have restricted ourselves to the distortion of trends caused by biases in the measurement. Trends caused by other reasons such as the economic crisis, although also interesting to investigate, fall therefore outside the scope of this paper.

### Time Series on Happiness and Satisfaction with Life in The Netherlands

The eldest time series on life satisfaction comes from the Eurobarometer (EB), a series of bi-annual public opinion surveys conducted in the member states of the European Union regularly on behalf of the European Commission, which dates back to 1973. The standard version of the EB has, almost without exception, a spring wave and an autumn wave for every year since then (Schmitt et al. 2008; European Commission 2012a, b, 2013). The number of waves mounted up to 76 in the period from 1973 to 2012, using the same 4-point verbal scale item every wave, except for wave 76.2 in the autumn of 2011 in which the answer to the same question had to be rated on a 10-point numerical scale. The EB was also the first survey used to measure happiness periodically. These measurements started in 1975 and were repeated every year until 1986 with interruptions in 1980 and 1981. There were 14 waves in total for this period, in all of which a 3-point verbal item was used. Happiness was also measured in version 66.3 of the EB in 2006. This was a special version of the EB in order to better understand the social realities of European Union citizens and a 4-point verbal scale was used to measure happiness. The measurement of happiness in this wave however, cannot be considered to be part of a time series, since in none of the other waves of the EB happiness was measured using the same scale.

The measurements with the EB were closely followed by a series of measurements by Statistics Netherlands (CBS) on life satisfaction in 1974 in The Netherlands in the first Life Situation Survey that CBS developed at the request of, and in close collaboration with, The Netherlands Institute for Social Research (SCP).^3^ Happiness followed in 1977. The 5-point verbal scale items for happiness and life satisfaction were used in changing surveys by both organisations and with different periodicities over a period of almost 40 years (DeJonge 2009). CBS used the item for happiness in 26 waves and the item for life satisfaction in 27 waves in the period from the first wave until 2009. After having conducted a split half experiment in 2012, in which a verbal and a numerical scale were used for both topics CBS decided to change to 10-point numerical response scales (van Beuningen et al. 2014). In the same time frame SCP had measured happiness 15 and life satisfaction 22 times using the verbal scales, but it changed to a 10-point numerical scale for life satisfaction in 2002.

Happiness has also been measured in the annual Dutch Household Survey (DHS)^4^ since 1993, resulting in 20 waves by 2012. In the 5 low frequency waves of the World Values Survey (WVS)^5^ carried out in The Netherlands since 1981, happiness has been measured using a 4-point verbal scale and life satisfaction is measured using a 10-point numerical scale. Finally, items on happiness and life satisfaction form part of the European Social Survey (ESS)^6^ which was fielded for the first time in The Netherlands in 2002 and since then, with a periodicity of 2 years. The ESS uses an 11-point numerical scale for both items and the results from 6 waves are available for the period 2002–2012. The aforementioned items constitute all the time series for happiness and life satisfaction in The Netherlands that we had to hand for this paper. An overview of these (translated) items is given in Tables 1 and 2, excluding the 4-point verbal scale used in wave 66.3 of the EB, the numerical scale used in wave 76.2 of the EB and the new items with a numerical scale of CBS. In both tables we have denoted the scale type as the number of response options followed by a p and an indication of whether it is a verbal scale or a numerical scale. For example, 3p-v denotes a 3-point verbal scale and 10p-n a 10-point numerical scale.

**Table 1:** Items used in time series of happiness in the Netherlands, 1975–2012

Survey	EB	WVS	CBS
Scale type	3p-v	4p-v	5p-v
Survey question	Taking all things together, how would you say things are these days? Would you say you are…?	Taking all together, to what extent do you think of yourself as a happy person?	To what extent do you consider yourself a happy person?
Response options	Very happy Pretty happy Not too happy	Very happy Quite happy Not very happy Not at all happy	Very happy Happy Neither happy nor unhappy Not very happy Unhappy

Survey	SCP	DHS	ESS
Scale type	5p-v	5p-v	11p-n
Survey question	To what extent do you consider yourself a happy person?	Taking all together, to what extent do you think of yourself as a happy person?	Taking all things together, how happy would you say you are?
Response options	Very happy Happy Neither happy nor unhappy Not very happy Unhappy	Very happy Happy Neither happy nor unhappy Unhappy Very unhappy	10 Extremely happy ⋮ 0 Extremely unhappy

**Table 2:** Items used in time series of life satisfaction in the Netherlands, 1973–2012

Survey	EB	CBS	SCP
Scale type	4p-v	5p-v	5p-v
Survey question	On the whole how satisfied are you with the life you lead?	To what extent are you satisfied with the life you currently lead?	To what extent are you satisfied with the life you currently lead?
Response options	Very satisfied Fairly satisfied Not very satisfied Not at all satisfied	Extraordinarily satisfied Very satisfied Satisfied Fairly satisfied Not very satisfied	Extraordinarily satisfied Very satisfied Satisfied Fairly satisfied Not very satisfied

Survey	SCP	WVS	ESS
Scale type	10p-n	10p-n	11p-n
Survey question	How satisfied are you with the life you currently lead?	All things considered, how satisfied are you with your life as-a-whole now…?	All things considered, how satisfied are you with your life as a whole nowadays?
Response options	10 Completely satisfied ⋮ 1 Completely dissatisfied	10 Satisfied ⋮ 1 Dissatisfied	10 Extremely satisfied ⋮ 0 Extremely dissatisfied

The diversity in *leading questions* and the corresponding response scales can clearly be seen from Tables 1 and 2. Some of the leading questions refer to life as whole framed, or not, in the present time, others just to refer to the life currently lead. The diversity in survey items however, is even greater in the number and labelling of the *response options*. The number of response options of the items listed in the two tables varies from three to eleven and the wording of the response options varies in many ways. This makes some scales unipolar, such as that of the EB item on life satisfaction in which the word ‘satisfied’ is used in the wording of all response options. Furthermore, this EB item is symmetric but has, in contrast to, for example the DHS item on happiness, no neutral middle option. Other scales have clearly defined anchor points of which the SCP item on life satisfaction is a good example, using the word completely in the labels of these points, which cannot be said of the asymmetric life satisfaction scale of the CBS item which has a response option at the lower end of the scale with a label that fails to express the lowest degree of satisfaction. These are just some examples of the variations in scales that are revealed when looking at the items in the two tables.

Note: an extensive overview of the ways in which survey items can differentiate can be found in Saris and Gallhofer’s (2007) book on survey design.

### The Problem of Incomparability of Time Series from Different Surveys

In survey research it is common practice to assign ranks to the response options of a discrete scale to calculate a sample mean, regardless of the wording used to label the options. The sample mean is accordingly calculated as the sum of the ranks weighted by the relative scores to each of the response options. In this common practice it is implicitly assumed that equivalent response options in equivalent scales on different topics are appreciated equivalently and that the response options are equally distanced. The degree of appreciation assigned to the words by which a response option is labelled, however, heavily depends on the context of the scale just as does the distance between two consecutive response options (DeJonge et al. 2014b). This notion emphasizes the difficulty of comparing the outcomes of different surveys.

**Fig. 1:**
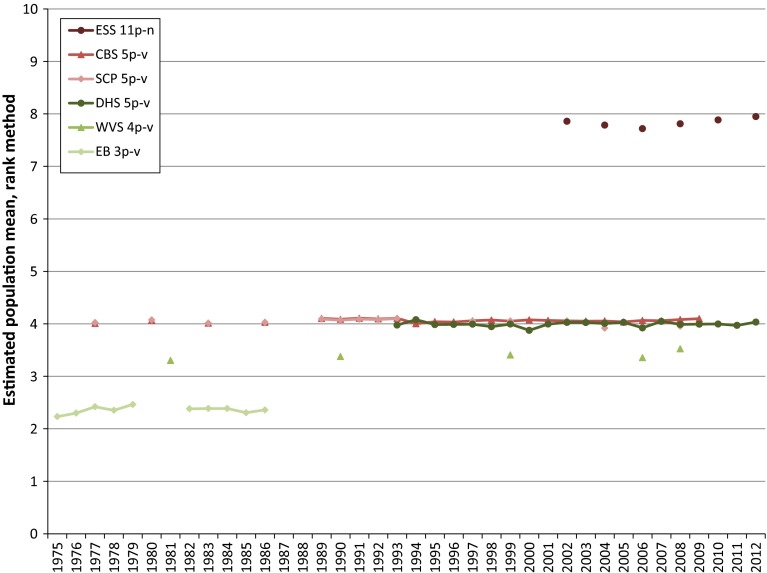
Mean happiness in The Netherlands based on ranks options primary scale

**Fig. 2:**
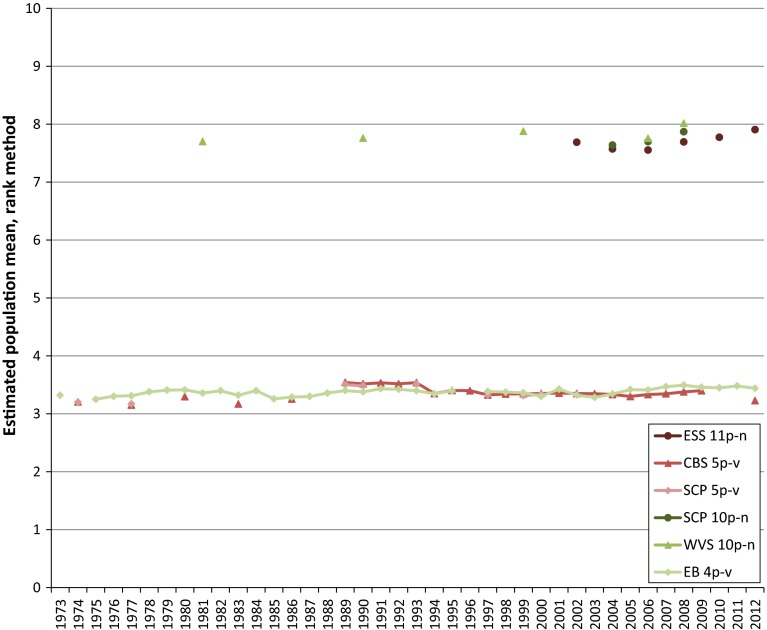
Mean life satisfaction in The Netherlands based on ranks options primary scale

There are a number of other problems for trend analyses that complicate the comparability of survey outcomes and it is difficult to pool these results into long, consistent time series. Much of this becomes clear if we look at Figs. 1 and 2, in which the time series of the sample means according to the common practice sketched above, denoted the rank method, are presented for the items given in Tables 1 and 2. Since for the EB item, for most years, there is more than one wave of measurements available, we calculated the un-weighted average of the frequency distributions for these items for each year to obtain one sample mean per year.

As can be seen from both figures it is obvious that there are considerable scale-effects if the rank method is applied to calculate a sample mean. From a quick glance, the effect of the difference in labelling may be not as obvious. Intuitively, the differences in sample means between a 4-point scale and a 5-point scale when applying the rank method will be more like those shown in Fig. 1, however, this is not the case for the sample means in Fig. 2. This is due to the difference in scales used between the surveys. This difference causes respondents who are satisfied with their life to select a response option with an, on average, relative high rank when they have to rate their life satisfaction on the 4-point EB scale, and to select a response option with an, on average, relatively low rank when they have to rate their life satisfaction on the 5-point CBS scale (DeJonge et al. 2014b). Another fact which has to be taken into account when improving the comparability of time series is the periodicity of the measurements which differs per survey and which has changed over the course of time for some surveys. This may also cause discontinuities, which is clearly visible from Figs. 1 and 2.

The above are just some of the issues that have to be addressed when searching for causes of incomparability, but they undoubtedly contribute to the understanding that the pooling of outcomes of different survey items into long consistent time series is not a straightforward exercise.

### Methods to Achieve Comparability of Survey Results

The limited uniformity of survey items and the other problems that affect the comparability of survey results reduces our ability to accumulate knowledge of such topic items and limits our analyses of trends. This calls for methods to transform ratings on different scales to attain comparable results and to correct for effects of changes in measurements and other influencing factors. Over the course of time a number of transformation methods have been developed. A comprehensive description of these methods is given in DeJonge et al. (2014a) which we briefly summarize in this paper. The simplest of the transformation methods is the Linear Stretch Method in which the ranks of the response options are stretched to meet a common range. This method is, for example, still applied in the World Database of Happiness for numerical scales with at least seven points and in the percentage of scale maximum (SM) method developed by Cummins (1997, 2003).

More advanced transformation methods deploy judges to rate the verbal labels of the response options on a common numerical scale. In one variation judges are asked to rate the degree of appreciation denoted by the labels of verbal response options on a common numerical scale. The average rating given to each label is kept fixed for every response scale of which it is part. The studies conducted by Jones and Thurstone (1955) and Lodge (1981) are illustrative of this variation. In a second variation judges assess points on a common, bounded continuum at which verbal response options for a given response scale transit from one to another (Veenhoven 2008). The mid-interval value between the two transition points of a verbal response is adopted as the secondary rating of this response option.

All these transformation methods have in common that they transform the response options on a primary scale to numbers on a secondary scale. The sample mean is calculated similar to common practice but with the ranks of the response options on the primary scale replaced by the transformed numbers. This sample mean is adopted as the estimator of the mean value of the distribution of, in our case, happiness or life satisfaction in the population. Although in these methods the primary scales of the different surveys are transformed to a common range, none of the methods overcomes the comparability problem. Suppose for instance that the results for life satisfaction in 2008 from one EB wave and those from CBS in the same year are transformed by applying the Linear Stretch Method, one would get a transformed mean of 5.9 for the EB item and of 8.2 for the CBS item (DeJonge et al. 2014a, pp. 284–285), whereas one would expect the transformed means to be the same since they are both taken from representative samples from the same population in the same year. Moreover, these methods are not suited to dealing with discontinuities in time series, and this also hampers the analyses of trends over time.

The Reference Distribution Method (DeJonge et al. 2014a) is a method which has been developed recently to tackle the shortcomings of the transformation methods mentioned above. The idea that underlies the Reference Distribution Method is that the estimated means of the response to equivalent questions about the same topic and asked in different representative surveys in one year should be approximately the same, irrespective of the primary response scales used. This method relies heavily on the Continuum Approach (Kalmijn 2010), in which happiness in the population is postulated to be a latent variable with a continuous probability distribution function. Within a specified ‘family’ of distributions, the one that is selected is the one that best fits the transitions points combined with the frequency distribution of the primary verbal response scale. Contrary to the transformation methods described previously the Continuum Approach is referred to as a non-transformation method, because in this approach no secondary ratings are introduced nor is there a relation between the corresponding parameters of the discrete sample distribution and the continuous one in the population.

### Research Question

The main question addressed in this paper is: Is the Reference Distribution suitable to use for pooling time series based on equivalent but not identical survey questions about the same topic? To answer this question we apply the method to pool the time series for happiness and life satisfaction in The Netherlands described in Sect. 1.2. As a by-product of this investigation we answered the question: Have the Dutch become more happy and satisfied with their lives over the past four decennia?

## The Reference Distribution Method

The idea that underlies the Reference Distribution Method is that the estimated means for equivalent questions about the same topic asked in different representative surveys in one year should be approximately the same, irrespective of the type of primary response scale used.

### Application of the Continuum Approach to Derive a Reference Distribution

The Reference Distribution Method (DeJonge et al. 2014a) is a method which relies heavily on the Continuum Approach of Kalmijn (2010). The main assumption of this approach is that variables are continuously distributed in a population. We quote from DeJonge et al. (2014c) that in the case of happiness a beta distribution is the most appropriate to use in the Continuum Approach due to at least three interesting properties it has (Kalmijn et al. 2011, pp. 509–510)

1. it is a continuous distribution, which makes it suitable as a model for the continuous latent happiness variable in the population
2. the random variable has a two-sided bounded domain, which makes it suitable for happiness as it is measured using two-sided bounded primary scales.
3. the distribution has two shape parameters, which makes beta distributions cover a wide class of different distribution shapes, including skew distributions, both positive and negative.

We do not know any other distributions with these properties. More generally known alternatives as the normal distribution and the logistic distribution are less suitable than the beta distribution, among other things because their domain is infinite and because they are bell-shaped and symmetric around their mean (Kalmijn 2012), whereas happiness has clearly skew distributions (Lee et al. 1982; Cummins 2003; Frijters et al. 2008; Guven et al. 2011).

The family of beta distributions consists of a series of distributions, each member of which is characterized by two shape parameters, *α* and *β*. The mean *μ* of a beta distribution with parameters *α* and *β* on the continuum from 0 to 10 is equal to:

1 $$\mu = 10\frac{\alpha }{\alpha + \beta }$$

The general formulae of the beta distribution and its probability density function and some examples of the probability density functions and the cumulative distribution functions for different values of *α* and *β* are given in Appendix 1.

A starting point for the Continuum Approach to determining national happiness is provided by the cumulative frequencies of measured happiness within a nation on a discrete primary scale and the values on a continuum from 0 to 10 at which respondents change their judgment from one to the adjacent response option on this primary scale, for example from ‘happy’ to ‘very happy’. In the Continuum Approach the shape parameters *α* and *β* of the beta distribution that fits best to the cumulative frequencies and the values on the continuum of the boundaries between the response options of the primary scale are estimated as maximum likelihood estimators. This estimation procedure is described into more detail in Kalmijn (2010, pp. 160–162).

There is always a perfect fit in the case of a primary scale with three response options. If the number of response options is restricted to only two, then there is no single solution: the number of perfectly fitting beta distributions is infinite, and use of the Continuum Approach is therefore invalidated. In the case of at least four response options, then in general there will be no perfectly fitting beta distribution and the best fitting solution should be taken. Those who are interested in the methodological considerations of the Continuum Approach are referred to Kalmijn (2010, Ch. VI) and Kalmijn et al. (2011).

If the Continuum Approach is used to derive a reference distribution based on survey results measured on a discrete scale, this scale should preferably be numerical with ten to eleven response options. This is in line with the assumption made by Voorpostel et al. (2009) that attitudes fall along a single, latent continuum and that the larger the number of points on a response scale, the better it represents this underlying, latent continuum and the more accurately it reflects the variation. Voorpostel et al. also state that ‘the larger the number of points, the more powerful the scale is in discriminating, but at a certain point respondents become unable to make fine distinctions and thus round off’. In addition, Saris and Gallhofer (2007, pp. 118–119) mention that respondents who are asked to give an answer on a magnitude scale with fixed reference points, which is comparable to a bounded continuum, have a tendency to prefer numbers which can be divided by five, leading to peaked response distributions. They state that this does not happen if line production scales^7^ are used, but that, due to practical considerations when using other modes of surveying, continuous scales may have a future once computer-assisted interviewing becomes more popular.

**Fig. 3:**
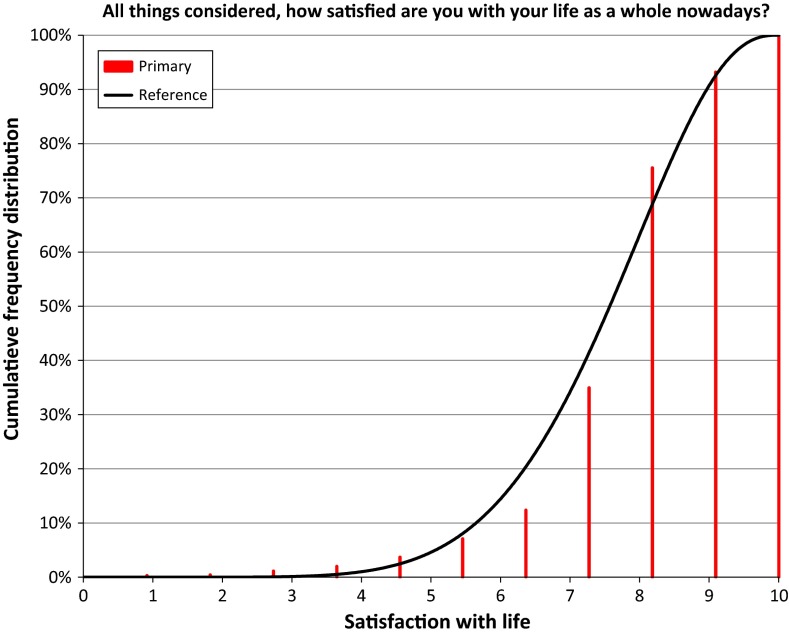
Best fitting beta distribution to the ESS frequency distribution 2008

In Fig. 3 we illustrate the use of the Continuum Approach to derive a reference distribution from responses to the question: ‘All things considered, how satisfied are you with your life as a whole nowadays?’ using an 11-point numerical scale from 0 to 10 with the anchor points of the scale labelled by ‘Extremely unsatisfied’ and ‘Extremely satisfied’. This item is in the core questionnaire of the European Social Survey (ESS).^8^ We fixed 11 equidistant upper boundaries, one for each response option, starting at 0.91 for the response option at the lower end of the scale and ending at 10.0 for the option at the upper end of the scale. The assumption that these boundaries between the response options are equidistantly distributed is a methodological choice (Kalmijn 2013) which provides a very useful basis for the Reference Distribution Method. We used the Continuum Approach to estimate the beta distribution that best fits these boundaries and the ESS life satisfaction cumulative frequency distribution for The Netherlands in 2008. The result is depicted in Fig. 3. We have drawn the cumulative frequency for each response option of the discrete primary ESS scale as a vertical bar at the position of the equidistant reference boundaries on the horizontal axis of Fig. 3. These cumulative frequencies are 0.3, 0.5, 1.1, 2.0, 3.7, 7.1, 12.4, 34.9, 75.5, 93.2 and 100.0 %. The curve in Fig. 3 is the beta distribution that according to the Continuum Approach fits best to the boundaries and cumulative frequencies distribution of the ESS item in 2008.

The parameters of the beta distribution in Fig. 3 are *α* = 7.92 and *β* = 2.76, which, according to Eq. 1 corresponds to a mean of 7.41.

### Illustration of an Application of the Reference Distribution Method

**Fig. 4:**
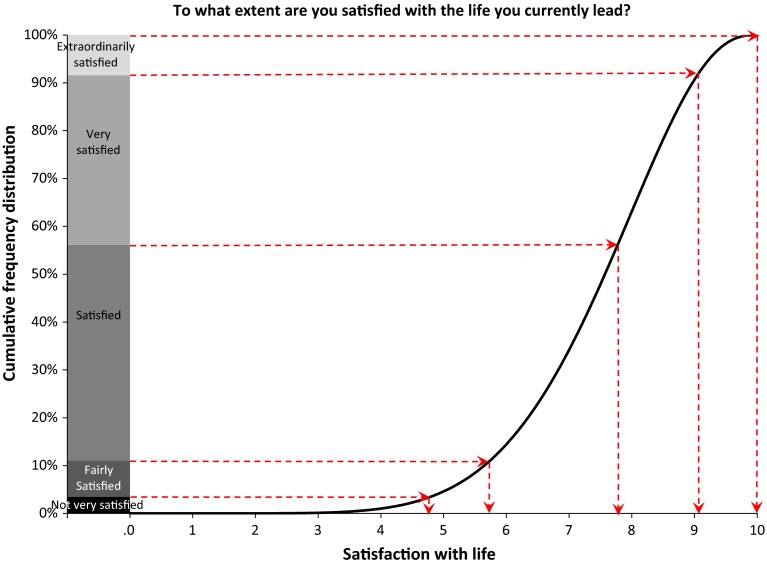
Illustration of the application of the reference distribution method to life satisfaction in The Netherlands in 2008

The best fitting beta distribution described in Sect. 2.1 serves as a reference for the application of the Reference Distribution Method to other response scales with an equivalent leading question and a frequency distribution for 2008. For each of these other scales the boundaries between the response options are determined by application of the Reference Distribution Method as the points where the cumulative frequency of the scale in the reference year matches with the reference distribution. We refer to the boundaries thus found as reference boundaries, since the reference distribution fits perfectly to these boundaries and the cumulative frequency distribution of each other scale in the reference year. The mean of the reference distribution is therefore an estimate of the mean on the 0-10 continuum for each of these other scales in the reference year. We illustrate how the reference boundaries for a response scale can be determined by applying the Reference Distribution Method using the reference distribution described in Sect. 2.1 to an item on life satisfaction taken from the Permanent Onderzoek Leef Situatie^9^ Survey of Statistics Netherlands (CBS) in Fig. 4. The leading question of this item is ‘To what extent are you satisfied with the life you currently lead?’ A 5-point verbal response scale is used to measure the level of satisfaction. The frequency distribution of the responses to this item in 2008 in The Netherlands is:

- Extraordinarily satisfied: 8.4 %
- Very satisfied: 35.5 %
- Satisfied: 45.1 %
- Fairly satisfied: 7.6 %
- Not very satisfied: 3.4 %

Using the Reference Distribution Method, the procedure to determine the reference boundaries between the response options of the CBS item on the continuum from 0 to 10 is as follows.

- We start with the cumulative frequency distribution of the CBS item for which we want to determine where the boundaries between the response options are positioned on the continuum from 0 to 10. This cumulative frequency distribution is shown as a stacked bar on the left side of Fig. 4.
- The reference distribution derived from the ESS is depicted to the right side of this stacked bar and plotted against the 0 to 10 continuum which is represented by the horizontal axis.
- A horizontal line is drawn from the cumulative frequency displayed in the stacked bar on the left side of Fig. 4 for each response option of the response scale to the point where it touches the reference distribution. At this point the value of the reference distribution is equal to the cumulative distribution on the scale of the CBS item.
- From this point down, a vertical line is drawn to the 0–10 continuum on the horizontal axis. The value at which the vertical line touches the horizontal axis is the position of the reference boundary of the corresponding response option.

Following this procedure, the reference boundaries for the response options of the CBS item on life satisfaction on the 0–10 continuum are, consecutively, 4.8, 5.7, 7.8, 9.0 and 10.0.

With the reference boundaries at these positions, the reference distribution perfectly fits to the cumulative frequency distribution of the CBS item in 2008. The mean 7.41 of the reference distribution is thus also an estimate of the population mean for 2008 wave of the CBS item. The reference boundaries can in their turn be used as input for the application of the Continuum Approach to the cumulative frequencies of the CBS item obtained in other waves. The estimated mean on the 0-10 continuum for each of these waves is equal to the mean of the corresponding best fitting beta distribution resulting from the application of the Continuum Approach. To exemplify this, the cumulative frequencies of each wave of the CBS item in the period 1997 to 2009 and the parameters and mean of the best fitting beta distributions are given in Table 5 of Appendix 2.

## Distortion of Trends Due to Biases in Measurement

A number of sources of measurement bias can be determined which may affect a respondent’s response to survey items and muddle the trend analyses by causing discontinuities. Some of these biases can be reduced using the Reference Distribution Method. Therefore a thorough inspection of a time series of data is necessary to determine possible biases that may occur and which have to be addressed before any application of the method to obtain a transformed time series.

### A Diversity of Sources of Measurement Bias

In Sect. 1 we introduced the issue of scale effects arising from differences in the numbers of response options, the labelling of the response options and whether a scale is numerical or verbal, however, something we did not mention above, is that the visual presentation of a scale can also influence results. This set of scale effects provided the main motivation and angle of approach for the development of the Reference Distribution Method and the preceding scale transformation methods commonly used in social research. All the methods used to date are aimed at overcoming the incomparability of survey results arousing from the scale effects. Further, if these methods are going to be used to transform time series to perform analyses of trends over a long time span, than a number of other sources of response bias must be addressed. Here we give an overview of these response biases in relation to the Reference Distribution Method. This overview is based on the description of these issues to be found in the book of the National Research Council (NRC) (2013, Ch. 4). In this book the sources of response bias we will deal with are, besides that of scale effects, mode of surveying, ordering of questions, response shift and contextual influences.

### Survey Mode Effects

Surveys can be conducted in a number of modes. Among these modes are face-to-face interviews, telephone interviews, paper- and-pencil surveying, surveying via the internet, or a mixture of the modes. Which mode is chosen, depends among others on the goal of the survey, the costs of surveying, or the size of the sample, and the time when, historically, the survey was conducted. The time of conducting the survey is of importance in relation to time series. Telephone interviews for example, only became possible at the time it was reasonable that almost all of the respondents would have a telephone at home, however, the rise of mobile phone usage has come at the cost of the adequate registration of telephone numbers, this causing this mode of questioning to become statistically invalid. The internet has opened up new opportunities for interviewing and its use is becoming ever more popular for survey research, not in the least for the relatively low costs associated with this mode of surveying.

The mode of surveying used has a large impact on a respondent’s response. In the Netherlands, for example, in 2004 SCP changed the mode of its Life Situation Survey from computer assisted personal interviews to a paper-and-pencil questionnaire. This caused a dramatic fall in the percentage of people who rated themselves as either ‘happy’ or ‘very happy’, a drop of 6 percentage points from 2002 to 2004 in a time series that had been rather stable from 1997 up to that point. Another example of the mode effect is described by Dolan and Kavetsos (2012), who found large differences in survey mode which swamped all the other bias effects that they had taken into account in their analysis.

As long as the mode of surveying is unchanged, the application of the Continuum Approach results for each wave in an estimated beta distribution that tightly fits to the reference boundaries derived from a reference distribution and the cumulative distribution of the primary scale. If the mode of surveying does not change, the reference boundaries can thus be kept fixed over time and the differences in estimated means can solely be attributed to changes in the frequency distributions on the primary scale (DeJonge et al. 2014c). If, however, a change of survey mode takes place, new reference boundaries must be derived which, on their turn, can be kept fixed as long as the new mode of surveying is used. The original reference distribution should not be used to derive new boundaries that comply with the new survey mode. Instead the best fitting beta distribution given the boundaries derived from the original reference distribution and the frequency distribution of the survey results in the year prior to or equal to that in which the mode was changed, should be selected as a new reference distribution. Whether a new reference distribution should be based on the survey results for the year the mode was changed or for the year prior to that, depends on whether a double measurement was done: in an ideal situation a survey will be fielded in both modes in the year of change to get insight into the effects of the mode change. In this case the new reference distribution can be based on the survey results for the same year the mode was changed. If, unfortunately, no double measurements are available, but the survey results show minor changes from year to year, as a proxy the best fitting beta distribution estimated for the year prior to the year the questionnaire mode was changed can be used.

### Ordering of Questions

The NRC (2013) mentions a number of studies in which the effect question ordering has on the response to a survey is discussed. They refer among others to a paper of Deaton (2012), who found a large effect of question ordering on the responses to a question on subjective life satisfaction. How a question was answered, was affected by, for example, whether or not respondents had to answer a question about a subject such as politics or unemployment before they assessed their own satisfaction with life. More in general it is acknowledged in the literature that preceding questions may affect how respondents interpret the meaning of survey items and that this effect may be significantly large (Saris and Gallhofer 2007; OECD 2013).

The bias in trend analyses caused by the effect the question ordering has on the survey response cannot be solved using the Reference Distribution Method if the order of questions is changed in different waves of one and the same survey, unless the size of the effect is comparable to that of a mode change and can be addressed as such. The Reference Distribution Method however, can deal with a difference in question ordering if this difference is present *between* surveys, but not in succeeding waves of each survey separately. This is because in the Reference Distribution Method the sample means of different surveys are forced to a level that is equivalent to that of the reference distribution, this causing the possible effect of a difference in question ordering between surveys to vanish.

### Response Shift

The term ‘response shift’ originates from a study by Howard et al. (1979) conducted to study to which extent participants changed their level of self-perceived dogmatism during a communication skills training. The effect of the training made the participants change their internal standards for their measurement of dogmatism. Researchers introduced the term ‘response shift’ to denote this kind of change. Since this time a growing number of studies on response shift have been conducted, most in the field of health care (e.g. Schwartz et al. 2013).

Within the field of subjective well-being the NRC (2013) summarizes response shift as a term that is used to characterize changes in reporting over time. They differentiate the observed differences over time in self-reports of well-being into two main streams. One reflects true changes in the quality-of-life assessments made by respondents as a result of adaptation to changed circumstances. The second reflects measurement errors associated with a respondent’s internal scale recalibrations. An example of this type of internal scale recalibration given by the NRC is the case described of a person with chronic pain who rates this pain on average at 7 on a scale from 1 to 10. After having experienced the more intense pain of a kidney stone, the same person lowered their rating of the unchanged chronic pain at 5. Thus, this person made an internal scale recalibration of how they rate chronic pain.

The NRC concludes that adaptation cannot be characterized as a process that occurs uniformly, because every person is unique and adapts differently to changing situations. In analyzing time series however, it is of interest to monitor if there are general upwards or downwards trends in target groups or subgroups, and in subjective well-being trends which reflect true changes in quality-of-life, but, as described by the NRC, it is not yet possible to decompose these trends into what are ‘true’ changes in quality-of-life and effects of scale recalibration. Use of the Reference Distribution Method does not contribute to solving this entanglement. It is very well possible that over the course of time a slow change in scale interpretation has evolved which means that on average the present population will appreciate the response options in a response scale differently than that of earlier times, which can also be considered as a type of scale recalibration. For an application of the Reference Distribution Method, in which we try to bring sample means from different surveys to a comparable level, we assume that if at present response options are appreciated differently than in the past, this will be the case for all the response scales under consideration, verbal and numerical.

### Contextual Influences

Contextual influences refer to the research instruments used and the external circumstances at the time of a respondent’s assessment for a self-report. Contextual influences of the research instrument can be brought back to the mode of surveying used and the ordering of questions as discussed above. There are manifold influential external circumstances that one can think of, such as the weather conditions at the time of the assessment, recent life events or breaking news of the day, but also the demographic characteristics of a respondent, such as age, level of education or cultural background and whether a questionnaire is computer mediated or not.

When analyzing trends one has to be aware of these contextual influences. This holds especially when studying trends over a long period of time as a consequence of which the sample of respondents in the earlier waves of the time series represent a different population than the sample of respondents in the more recent waves. In an aging society for example, an increasing trend in life satisfaction might be due to a positivity effect of an age-related trend of the older person having a positive view towards life (NRC 2013). In another example, the composition of the population may change over the years due to migration movements, introducing an effect of cultural influences into the outcome of happiness measurements (Senik 2013). The above examples serve to illustrate how contextual influences in general affect trend analyses, independent of the response scales used. The Reference Distribution Method can be used appropriately to take a limited number of the possible contextual influences into account. If, for example, a survey is always conducted in the autumn or spring, than there may be a seasonal effect in the response to each wave, also, when an exceptional influential event takes places during the period of surveying the results may be biased. These types of contextual influences are, to a certain extent, corrected when the Reference Distribution Method is used, because the sample means of the surveys they belong to are shifted to a level that is equivalent to that of the reference distribution.

## Inspecting the Available Time Series

We have argued that when using the Reference Distribution Method it is possible to correct for discontinuities due to mode changes and, to a limited extent, for contextual influences. This requires a thorough inspection of the available time series, and decisions have to be made as to which waves require new reference boundaries to be derived and which estimated beta distributions can serve as a reference for these decisions. In addition we have to decide whether some waves have to be excluded from a transformation and for what valid reason.

### Searching for Sources of Response Bias to Correct

#### The CBS Items on Happiness and Life Satisfaction

The surveys which include the CBS items on happiness and life satisfaction have been changed several times since their introduction. These changes have affected the responses and led to serious discontinuities in the time series. In the first years the survey containing the items was only fielded over a period of a few weeks, changing to a continuous survey from 1989 on. The effect of this is undoubtedly visible, especially for the life satisfaction item in Fig. 2. A comprehensive revision of the questionnaire forms and a reduction in the number of survey items in several domains in 1994 led once more to a response discontinuity. Another major change in the CBS survey took place in 1997 when among other things the survey mode was changed from paper-and-pencil surveying into face-to-face interviews. This change was introduced using a split half measurement with half of the respondents being required to fill in a paper-and-pencil questionnaire and the other half being interviewed. In the most recent change to the CBS survey, in 2012, 10-point numerical scales were introduced, breaking with the previous tradition of using verbal scales (van Beuningen et al. 2014). Thus we left the 2012 wave of the CBS out of the conversion process, since as yet it provides only a singular point in the data flow.

#### The SCP Items on Happiness and Life Satisfaction

Much of which has been said for the CBS items also holds for the SCP items. In addition, as we mentioned in Sect. 3.2, SCP changed the mode of its Life Situation Survey, which included the item on happiness, from a computer assisted personal interview to a paper-and-pencil questionnaire in 2004. With the exception of the Life Situation Survey, SCP has included the life satisfaction item also over a long period in its survey of Cultural Changes in The Netherlands. The response to this survey is yet un-weighted and we therefore left it out of the conversion process. Note: in 2004, for rating life satisfaction SCP changed from using its traditional 5-point verbal scale to a 10-point numerical scale.

#### The Eurobarometer (EB) Items on Happiness and Life Satisfaction

Happiness was measured in the first years of the EB until 1986 and not in later years. In the waves of the EB from before 2002 the questions on life satisfaction and before 1987 also on happiness, were preceded by a number of opinion questions on different topics. In almost all waves of the EB from 2002 on, the question on life satisfaction is the first subjective question asked. We have seen no noticeable change due to this difference in question ordering between older and newer waves. The main point of concern with respect to the EB is the fact that there is a spring and a summer wave in each year and in some years one or more extra waves. This may be the reason why the individual waves of the time series of the EB show a rather irregular pattern, which may be due to by seasonal effects. For this reason, but also because we preferred to have one measurement a year, we averaged the response to all waves per year for the EB.

#### The DHS Item on Happiness

In the time series of the DHS item on happiness there are noticeable discontinuities in the waves of 2000 and 2006. We have no information about the background of these discontinuities and therefor decided to leave them as they are.

#### The WVS Items on Happiness and Life Satisfaction

The WVS items have been fielded with large gaps in the major series. Despite this, we had no reason to assume that there is a need to correct for discontinuities, except for the item on happiness for the wave of 2008. Compared to the wave of 2006, the frequency of respondents who rated themselves as very happy increased from 42 to 56 %. We do not see such a large increase in any of the other surveys carried out in the same period and therefore considered this result of the WVS for 2008 to be implausible and left it out of the conversion process.

### Preparation for the Conversion of Time Series

The ESS is the only recent survey available in which both happiness and life satisfaction are measured on an 11-point numerical scale. The ESS is therefore an obvious choice to select when estimating initial reference distributions. The ESS has been fielded every 2 years since 2002. We estimated a best fitting beta distribution to the frequency distributions of each wave of the ESS, keeping the numerical response options equidistant. In this way we got a conversion of the time series from the ESS into a series with fits to the 0–10 continuum.

Because most of the time series from other surveys we have at our disposal contain a wave for 2008, this became the best choice to serve as the initial reference year. Starting with 2008, we began the conversion process for the other surveys to convert the responses from waves of 2008 and before, but we also converted the responses from more recent waves. The exception to this was the happiness item of the WVS, for which we derived reference boundaries from the beta distribution estimated for the ESS-results over 2006, due to having excluded the WVS over 2008 from the conversion process for the reasons discussed above.

**Fig. 5:**
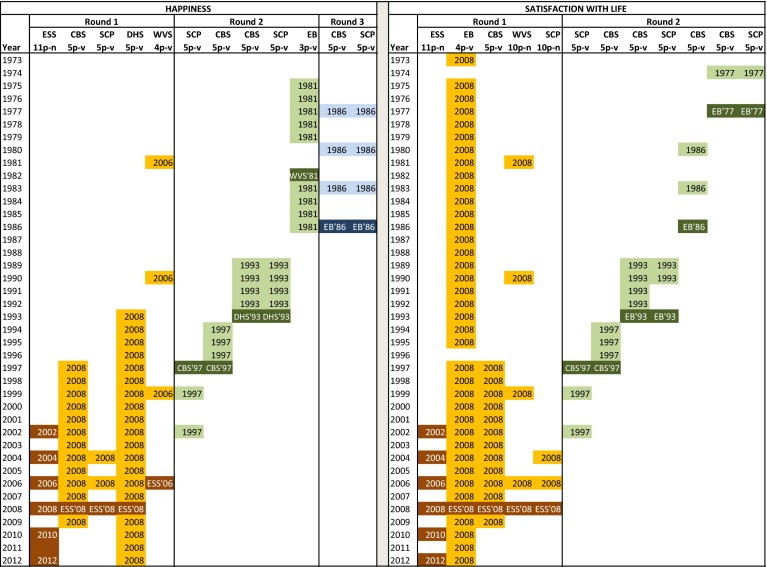
Conversion schemes happiness and life satisfaction

We have made conversion schemes for the time series for happiness and life satisfaction to prepare and support the conversion process. These schemes are shown in Fig. 5 in which the waves for the ESS items are indicated by the corresponding year on the left side of each conversion scheme. It can be seen in Fig. 5 that several rounds were needed for the conversion process. This is a consequence of the discontinuities in the time series of some surveys and of the fact that not all surveys were in use in the entire period spanned by all the time series together. The dark coloured boxes with text in white in Fig. 5 represent a year and survey item for which a beta distribution is estimated that is used to derive reference boundaries from for other survey items. The reference boundaries derived for each of those other surveys on their turn are used to apply the Continuum Approach for estimating a best fitting beta distribution for each of the other waves in the corresponding column. This is indicated, for example, for the waves of the CBS life satisfaction item in the period 1997–2009 by the text ‘2008’ in Fig. 5.

From Fig. 5 it can be seen that we needed to derive reference boundaries for a number of items and a number of years. The reference boundaries for the 1982 wave of the EB item were derived from the estimated beta distribution for the 1981 wave of the WVS, which means that there was a one year difference between the waves from both surveys. This was a choice we made, because otherwise we would not have been able to convert the EB time series and the results from the older waves of the CBS and SCP time series.

We have presented the reference boundaries we derived in each round of the conversion process for happiness in Table 3 and similarly, the reference boundaries for life satisfaction are contained in Table 4. We have included the parameters α and β and the estimated population mean for each reference distribution used.

**Table 3:**
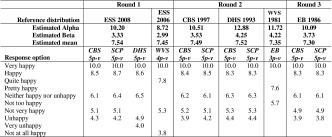
Reference distributions and reference boundaries for happiness items

**Table 4:**
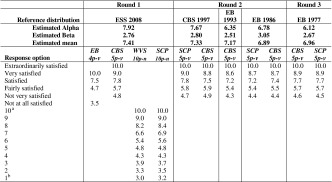
Reference distributions and reference boundaries for life satisfaction items

^a^In the WVS labelled with ‘Satisfied’ and in the SCP survey labelled with ‘Completely satisfied’

^b^In the WVS labelled with ‘Dissatisfied’ and in the SCP survey labelled with ‘Completely dissatisfied’

It can be seen from Table 3, that reference boundaries for different waves of one time series do not differ much. For response options with the same label but in different scales, the differences are much larger, which is most notably for the response options labelled with ‘Unhappy’. This corroborates the idea that how a response option with a certain label is appreciated depends on the context of the scale (DeJonge et al. 2014b).

Two of the items included for life satisfaction have a 10-point numerical scale. The reference boundaries for the numerically labelled response options of these scales are clearly not equidistant as can be seen in Table 4. Despite the differences in the labels used for the anchor points of both scales, the reference boundaries do not differ much.

## Combining Converted Survey Results into Long Time Series

The beta distributions estimated for the time series of the different survey items for happiness and life satisfaction can be used to calculate estimates of the population means which are on a comparable level. Once this is achieved, the converted times series can be pooled into long consistent times series for happiness and life satisfaction in The Netherlands, spanning a period of almost forty years.

### Conversion Population Means for Time Series of Individual Survey Items

**Fig. 6:**
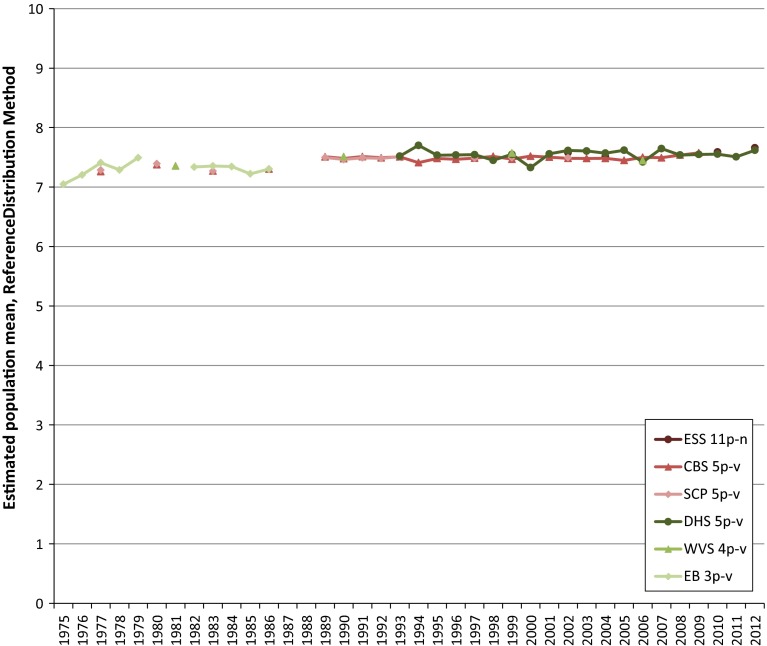
Converted time series for happiness in The Netherlands

**Fig. 7:**
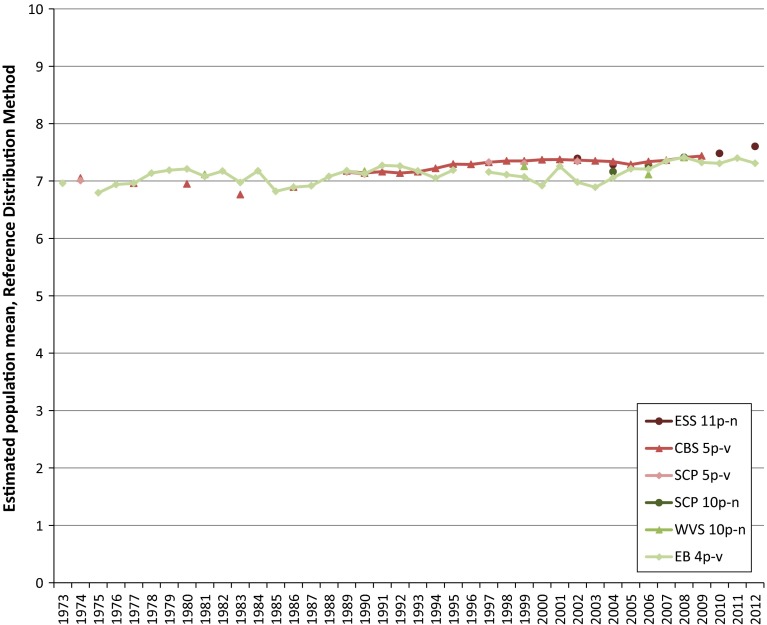
Converted time series for life satisfaction in The Netherlands

Given estimates of the parameters α and β of the beta distribution of happiness and life satisfaction, we can calculate an estimate of the population mean as (10*α/(α + β)) for each survey in a time series. We have exemplified this previously in Table 5 of Appendix 2. By doing this for all estimated beta distributions, we obtain a time series of converted population means for each individual survey item. We recall from Sect. 3.2 that if the mode of surveying does not change, the reference boundaries can be kept fixed over time when applying the Continuum Approach and the differences in estimated means can solely be attributed to changes in the frequency distributions on the primary scale. The use of reference distributions brings the means for different survey items to a comparable level. The time series of the converted means are presented in Fig. 6 for happiness and in Fig. 7 for life satisfaction.

Comparing the converted time series given in Figs. 6 and 7 with the unconverted time series in Figs. 1 and 2, it can be seen that the conversion process, as intended, brought the estimated population means to a comparable level, also, the pattern over the year for each individual time series remains largely unchanged after the conversion, except for the waves for where we corrected for discontinuities and for the magnitude of the fluctuations. The magnitude of the fluctuations is stretched somewhat, due to the conversion of the data from a low number of response options to a continuum spanning a larger numerical range.

### Pooling of Converted Time Series

**Fig. 8:**
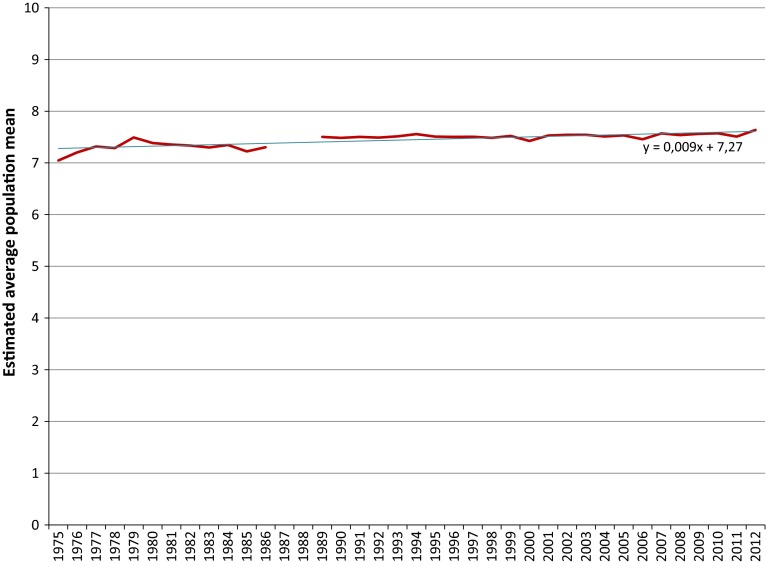
Pooled converted time series for happiness in The Netherlands

**Fig. 9:**
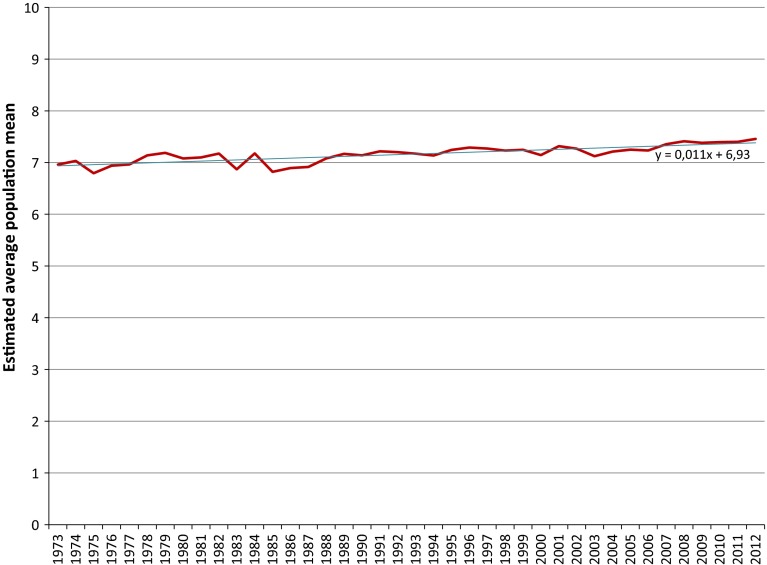
Pooled converted time series for satisfaction with life in The Netherlands

The last step to obtain one long time series for happiness and life satisfaction is to pool the converted time series of the individual survey items. We have chosen to pool the time series in the most straightforward manner one can think of, which corresponds to taking the average of the estimated population means for each year. This gives one un-weighted average population mean per year joining together all individual converted time series into the required time series. We have depicted these pooled time series in Figs. 8 and 9 to which we have also added the trend line to give an indication of the trends in happiness and life satisfaction in The Netherlands over the past 4 decennia.

It can be seen from Figs. 8 and 9 that pooling the converted time series has flattened the fluctuations of the individual time series. We conclude that the Dutch population has become slightly happier and more satisfied with life since the early seventies of the twentieth century. According to the trend lines, average happiness has increased by a little more than 0.3 points from 7.27 in 1975 to 7.60 in 2012, whereas the increase in average life satisfaction in the same period has amounted to a little more than 0.4 points going from 6.95 in 1975 to 7.37 in 2012.

## Discussion

In this paper we present an example application of the recently developed Reference Distribution Method for pooling time series based on slightly different survey questions on the same topic. We use the method to pool time series for the topics happiness and satisfaction with life. In this section we will discuss some methodological considerations and ideas for further research.

### Methodological Considerations

The standard version of the Eurobarometer has a spring wave and an autumn wave and sometimes one or more extra waves in a year. For the pooling of time series as described in this paper, we calculated the un-weighted average of the frequency distributions for each year of the Eurobarometer to obtain one sample mean per year. Another option would have been to consider the spring waves and the autumn waves each as constituting different time series and to convert them separately. In this case, the possible seasonal effect would not have been averaged out before the application of the Reference Distribution Method, but after the conversion when pooling them together with other survey time series into one long time series. It would then be possible to investigate whether or not the seasonal effect is more or less stable over time.

There are sizeable differences in the number of respondents per survey to the survey items we looked at. In pooling the converted time series, we did not take these differences into consideration and calculated an un-weighted average for each year. It would be interesting to investigate if the trends found would differ very much if, when averaging over the estimated means in a year, these means were weighted by the number of respondents to the corresponding survey.

We only used one wave of the ESS to estimate an initial reference distribution for the conversion of the time series of one survey. The initial reference distribution defines the level of reference for the conversion results of all waves from each survey. To obtain a more stable pooled time series that is less prone to systematic errors, it would be useful to consider repeating the whole conversion process using each wave of the ESS once to derive an initial reference distribution from. Instead of pooling time series which are converted on the basis of just one initial reference distribution, conversion results for initial reference distributions based on other waves could be included in the pooling.

We kept the boundaries between response options that were derived from a reference distribution fixed for all subsequent waves for which the survey design had not undergone a significant change. It is a reasonable assumption that the boundaries will be more or less stable over time and may be kept fixed as we have discussed in DeJonge et al. (2014c).

### Limitations of the Reference Distribution Method

The Reference Distribution Method can be used to correct for discontinuities due to mode changes and, to a limited extent, to account for contextual influences; yet it cannot solve all the comparability problems. For example an effect on the response due to a re-ordering of questions cannot be corrected for by the Reference Distribution Method if the effect is small or the re-ordering occurs frequently and affects the response of a number of waves. Furthermore, if there is a response shift, this will be difficult to correct for since in time series this shift may occur over a long period of time and we assume that if it occurs, this will be the case for all response scales under consideration, both verbal and numerical.

If a survey has been fielded only once and there is a reference distribution available, then the converted mean according to the Reference Distribution Method is, by definition, equal to the mean of this reference distribution. This saddles the converted scores with the errors of the reference distribution.

The Reference Distribution Method has been developed to be applied to single item questions. Yet, there are also multiple question inventories, such as Diener et al. (1985) five item ‘satisfaction with life scale’. Although each of these items can be tuned in principle, the chance of finding good reference items is lower than that for the case of single items.

### Issues for Further Research

The items used for the results in this paper, all come from surveys conducted in The Netherlands: to further improve and validate the method it is necessary to apply the method to time series of survey results from other countries, which at the same time will allow us to study the differences between countries in the interpretation of scales and how respondents in practice cope with response options.

So far the Reference Distribution Method has only been applied to time series for happiness and life satisfaction. It would be interesting to apply it to other topics related to the perceptions of people,^10^ to be able to compare long term trends for these topics. This would also give us an opportunity to find out whether or not using the family of beta distributions is appropriate for these topics when converting time series.

The pooled time series over the period 1989 to 2012 are uninterrupted for happiness and life satisfaction. A summary of the main statistics of the estimated mean value distributions for this period is given in Table 6 of Appendix 3. A comparison for this period shows that the pooled time series for both happiness and life satisfaction have an increasing tendency, but that the average annual increases are clearly unequal: 0.010 on a 0–10 continuum for life satisfaction and only 0.003 for happiness. As a consequence the difference in means between the two topics decreases on average by 0.007 per year. This might be an indication that the scientific definition of ‘happiness’ as ‘the subject enjoyment of one’s life as a whole’ is more and more understood as such in common language. This hypothesis could be tested on trends in other countries, but also in trends within subgroups of the population having experienced certain life events part of which is usually more associated with happiness than with life satisfaction and vice versa. If these tests require the pooling of responses to slightly different survey questions, than the method presented in this paper can be applied.

In the pooled converted time series for both happiness and life satisfaction, a growing tendency coincident with the economic crisis can be observed for the last years of analysis. This tendency in the pooled time series is a direct consequence of the trends in the time series based on the scores on the primary scales as can be noted from Figs. 1 and 2. It falls outside the scope of this paper to give an explanation for this tendency for happiness and life satisfaction in The Netherlands. Since it is a rather remarkable tendency however, it would be worthwhile to investigate what the tendency in the period of the economic crisis in other countries is.

Most of the points we mentioned under ‘Methodological considerations’ give rise to a need for more methodological research.

## Conclusions

We conclude that the Reference Distribution Method is a useful method to pool time series from different surveys to make the analyses of trends over a long time span possible. Having applied this method to pool time series of responses to different survey items on happiness and life satisfaction in the Netherlands, we conclude that in the past 40 years the Dutch have become slightly happier and more satisfied with their lives.

## Figures and Tables

**Fig. 10:**
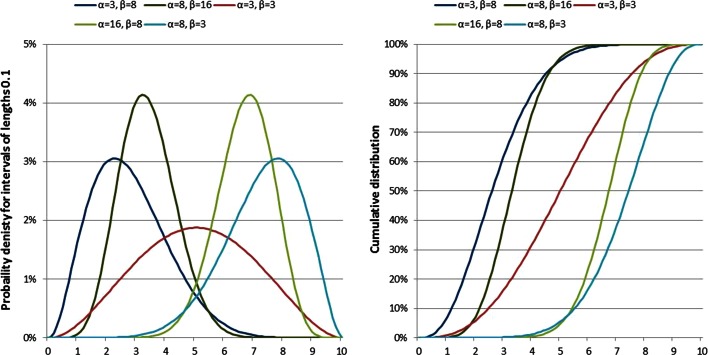
Examples of the probability density β-functions and cumulative distribution β-functions

## Appendix 1: The Beta Distribution and Probability Density Function

A beta distribution can be expressed using the complete beta function:

2 $$B\left( {\alpha ,\beta } \right) : = \mathop \int \limits_{0}^{1} t^{\alpha - 1} \left( {1 - t} \right)^{\beta - 1} dt$$

Given the formula (Eq. 2) the probability density function of the beta distribution on the continuum from 0 to 10 can be written as:

3 $$f(x|\alpha ,\beta ): = \left\{ {\begin{array}{*{20}l} {[10B(\alpha ,\beta )]^{{ - 1}} x^{{\alpha - 1}} (10 - x)^{{\beta - 1}} } \hfill & {{\text{for}}\,x \in [0,10]} \hfill \\ 0 \hfill & {{\text{otherwise}}} \hfill \\ \end{array} } \right.$$

Some examples of the probability density functions and the cumulative distribution functions for different values of *α* and *β* are given in Fig. 9.

If *α* < *β*, the probability density function is skewed to the right, if *α* > *β* the function is skewed to the left and if both parameters are equal the function is symmetric about *x* = 5. Furthermore, the larger the values of *α* and *β*, the more peaked the density curve and the steeper the cumulative distribution curve are (Fig. 10).

## Appendix 2

See Table 5.

**Table 5:** Cumulative frequencies life satisfaction item CBS and parameters and mean best fitting beta distributions to the reference boundaries 4.8, 5.7, 7.8, 9.0 and 10.0

Year	“To what extent are you satisfied with the life you currently lead?”					Parameters and mean best fitting beta distribution		
	Not very satisfied (%)	Fairly satisfied (%)	Satisfied (%)	Very satisfied (%)	Extraordinarily satisfied (%)	α	β	Mean
1997	4.1	12.4	58.7	92.5	100.0	7.69	2.81	7.33
1998	3.8	11.9	58.1	92.4	100.0	7.83	2.82	7.35
1999	4.0	12.1	57.9	92.1	100.0	7.69	2.77	7.35
2000	3.8	11.7	57.4	92.0	100.0	7.79	2.78	7.37
2001	3.7	11.6	57.2	92.0	100.0	7.80	2.78	7.38
2002	4.1	12.3	57.2	91.5	100.0	7.41	2.66	7.36
2003	4.2	12.4	57.6	91.7	100.0	7.46	2.69	7.35
2004	4.0	12.3	58.2	92.3	100.0	7.66	2.78	7.34
2005	4.4	13.2	59.7	92.8	100.0	7.55	2.81	7.29
2006	3.9	12.1	58.4	92.5	100.0	7.80	2.83	7.34
2007	3.7	11.6	57.8	92.4	100.0	7.94	2.84	7.36
2008	**3.5**	**11.0**	**56.1**	**91.6**	**100.0**	**7.92**	**2.76**	**7.41**
2009	3.9	11.6	54.7	90.0	100.0	7.24	2.50	7.44

The parameters and mean of the reference distribution and the frequency distribution for 2008 that has been used to derive the reference boundaries for the response options of the scale are given in bold

See also Table 12 in DeJonge et al. (2014c)

## Appendix 3: Statistics Mean Value Distributions Pooled Time Series

See Table 6.

**Table 6:** Main statistics of the estimated mean value distributions of the pooled time series in the period 1989–2012

Statistic	Happiness	Life satisfaction	Difference
Number of years	24	24	24
Maximum	7.64	7.46	0.42
Minimum	7.42	7.12	0.11
Range	0.21	0.34	0.31
Average	7.52	7.26	0.26
Standard deviation	0.043	0.097	0.079
Average annual increase	0.003	0.010	−0.007

## List of abbreviations

3p-v: 3-point verbal scale
4p-v: 4-point verbal scale
5p-v: 5-point verbal scale
10p-n: 10-point numerical scale
11p-n: 11-point numerical scale
EB: Eurobarometer
CBS: Statistics Netherlands (In Dutch: Centraal Bureau voor de Statistiek)
DHS: Dutch household survey
ESS: European social survey
NRC: National Research Council
OECD: Organisation for Economic Cooperation and Development
SCP: The Netherlands Institute for Social Research (In Dutch: Sociaal Cultureel Planbureau)
SM: Scale maximum
WVS: World values survey

## Footnotes

1 We use the term ‘anchor points’ for the response options at both ends of a discrete scale. In case of a continuous distribution, we use the term ‘extremes’ to refer to the boundaries of the range.

2 This paper is the fourth in a series of papers in which we focus on the comparability of responses to different survey questions on the same topic.

3 The frequency distributions of the items from CBS and SCP were obtained by personal communication.

4 CentERdata—Institute for data collection and research, www.dhsdata.nl.

5
http://www.worldvaluessurvey.org/wvs.jsp.

6
http://www.europeansocialsurvey.org/.

7 For line production scales respondents are asked to draw a line with a length that expresses the ratio of their judgment to the length of a standard line that is used as a reference judgment for a certain topic.

8
http://www.europeansocialsurvey.org/.

9 Permanent Survey on Living Conditions.

10 Other topics are for example how satisfied people are with their housing, their working conditions or their present job, but also how safe they feel when walking alone in their neighbourhood at night and how they assess their own health.
